# 1-Benzyl-1*H*-benzotriazole 3-oxide monohydrate

**DOI:** 10.1107/S1600536812044868

**Published:** 2012-11-07

**Authors:** P. Selvarathy Grace, Samuel Robinson Jebas, B. Ravindran Durai Nayagam, Dieter Schollmeyer

**Affiliations:** a Department of Chemistry, Popes College, Sawyerpuram 628 251, Tamilnadu, India; b Department of Physics, Sethupathy Government Arts College, Ramanathapuram 623 502, Tamilnadu, India; c Institut für Organische Chemie, Universität Mainz, Duesbergweg 10-14, 55099 Mainz, Germany

## Abstract

In the title hydrate, C_13_H_11_N_3_O·H_2_O, the benzotriazole ring system is planar (r.m.s. deviation = 0.007 Å) and is almost orthogonal to the phenyl ring to which it is linked by a methyl­ene group, forming a dihedral angle of 81.87 (15)°. In the crystal, mol­ecules are linked into chains along [001] by O—H⋯O hydrogen bonds. The chains are consolidated into a three-dimensional architecture by C—H⋯O, C—H⋯π and π–π [centroid–centroid distance between the five- and six-membered rings of the benzotriazole ring system = 3.595 (3) Å] inter­actions.

## Related literature

For the biological activity of benzotriazole derivatives, see: Kopańska *et al.* (2005 ▶); Sarala *et al.* (2007 ▶). For their applications, see: Kopec *et al.* (2008 ▶); Krawczyk & Gdaniec (2005 ▶); Smith *et al.* (2001 ▶); Sha *et al.* (1996 ▶). For a related structure, see: Selvarathy Grace *et al.* (2012 ▶).
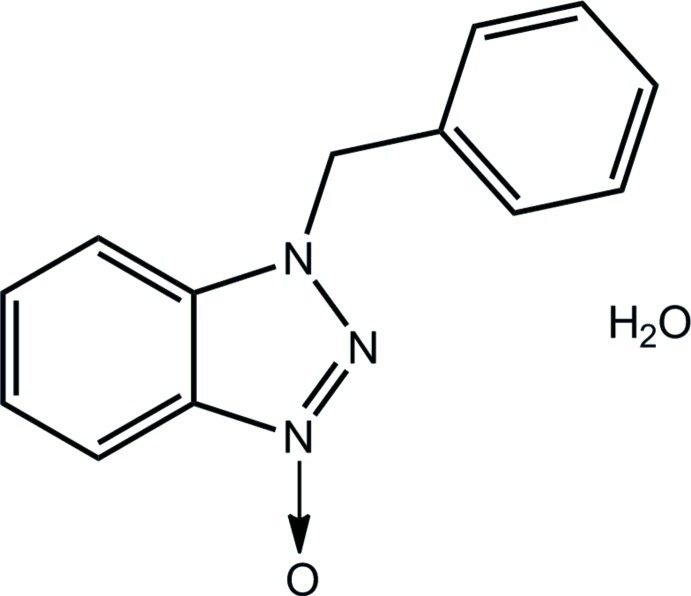


## Experimental

### 

#### Crystal data

C_13_H_11_N_3_O·H_2_O
*M* *_r_* = 243.26
Orthorhombic, 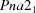

*a* = 12.556 (5) Å
*b* = 20.881 (8) Å
*c* = 4.6651 (18) Å
*V* = 1223.1 (8) Å^3^
*Z* = 4
Mo *K*α radiation
μ = 0.09 mm^−1^
*T* = 173 K
0.40 × 0.05 × 0.04 mm

#### Data collection

Bruker SMART APEXII diffractometer
15911 measured reflections
1677 independent reflections
1118 reflections with *I* > 2σ(*I*)
*R*_int_ = 0.132

#### Refinement

*R*[*F* ^2^ > 2σ(*F* ^2^)] = 0.052
*wR*(*F* ^2^) = 0.140
*S* = 0.98
1677 reflections
164 parameters
1 restraint
H-atom parameters constrained
Δρ_max_ = 0.19 e Å^−3^
Δρ_min_ = −0.24 e Å^−3^

### 

Data collection: *APEX2* (Bruker, 2008 ▶); cell refinement: *SAINT* (Bruker, 2008 ▶); data reduction: *SAINT*; program(s) used to solve structure: *SHELXS97* (Sheldrick, 2008 ▶); program(s) used to refine structure: *SHELXL97* (Sheldrick, 2008 ▶); molecular graphics: *SHELXTL* (Sheldrick, 2008 ▶); software used to prepare material for publication: *PLATON* (Spek, 2009 ▶).

## Supplementary Material

Crystal structure: contains datablock(s) global, I. DOI: 10.1107/S1600536812044868/tk5165sup1.cif

Structure factors: contains datablock(s) I. DOI: 10.1107/S1600536812044868/tk5165Isup2.hkl

Supplementary material file. DOI: 10.1107/S1600536812044868/tk5165Isup3.cml

Additional supplementary materials:  crystallographic information; 3D view; checkCIF report

## Figures and Tables

**Table 1:** Hydrogen-bond geometry (Å, °) *Cg*1 is the centroid of the C11–C16 phenyl ring.

*D*—H⋯*A*	*D*—H	H⋯*A*	*D*⋯*A*	*D*—H⋯*A*
O1*W*—H1*W*⋯O1*W* ^i^	0.82	1.93	2.744 (3)	169
O1*W*—H2*W*⋯O17	0.85	1.95	2.800 (3)	180
C10—H10*A*⋯O17^ii^	0.99	2.45	3.400 (5)	161
C10—H10*B*⋯*Cg*3^iii^	0.99	2.51	3.382 (4)	147

Symmetry codes: (i) 
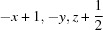
; (ii) 
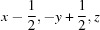
; (iii) 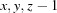.

**Fig. 1.**
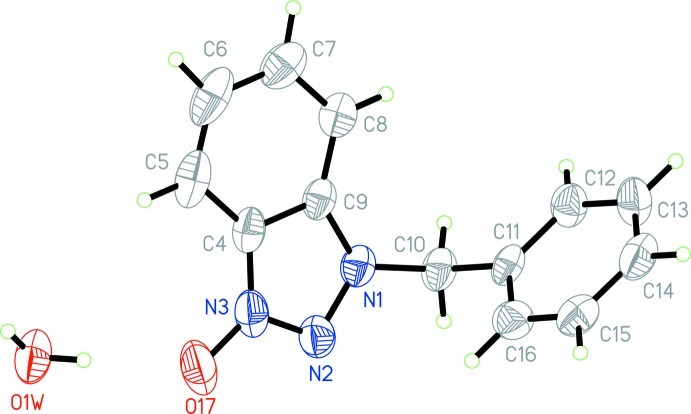
The asymmetric unit of the title compound, showing the atomic numbering scheme. Displacement ellipsoids are drawn at the 50% probability level.

**Fig. 2.**
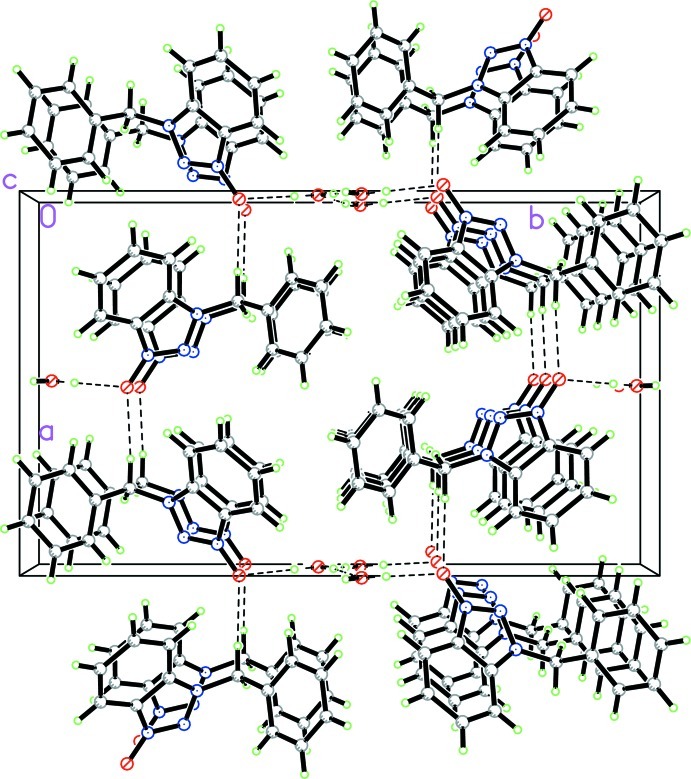
The crystal packing of the title compound, highlighting the one-dimensional chains along [001]. Hydrogen bonds are shown as dashed lines.

## supplementary crystallographic information

### Comment

Benzotriazole derivatives show biological activities such as anti-inflammatory, diuretic, anti-viral and anti-hypertensive (Kopańska *et al.*, 2005; Sarala *et al.*, 2007). They have been used as a corrosion inhibitor, anti-freeze agent, ultraviolet light stabilizer for plastics and as an anti-foggant in photography (Krawczyk & Gdaniec, 2005; Smith *et al.*, 2001). *N*-aryloxy derivatives of benzotriazole have anti-mycobacterial activity (Kopec *et al.*, 2008). Benzotriazole possessing three vicinal N atoms, is used as an anti-fouling and anti-wear reagent (Sha *et al.*, 1996). Due to the above mentioned applications of benzotriazole, we have systematically synthesised and investigated the structures of novel benzotriazole derivatives. We have already reported the crystal structure of 1-(benzyl)-1*H*-benzotriazole (Selvarathy Grace *et al.*, 2012). Here, we report the crystal structure of the title compound (I).

The benzotriazole ring in (I), Fig 1, is essentially planar with the maximum deviation from planarity being 0.010 (3) Å for atom N3. The mean plane of the benzotriazole ring (N1–N3,C4–C9) forms a dihedral angle of 81.87 (15) Å with the mean plane of the phenyl ring (C11–C16).

The molecules are linked into a one dimensional chain along [001] by O—H···O hydrogen bonds, Table 1 and Fig. 2. The crystal packing is stabilized by π–π stacking interactions with the centroid-centroid distance of 3.595 (3) Å [symmetry code: *x*, *y*, -1+*z*], together with C—H···O and C—H···π interactions, Table 1.

### Experimental

A mixture of sodium salt of 1-hydroxyl benzotriazole (0.157 g, 1 mmol) and benzyl chloride (0.126 g, 1 mmol) in a mixture comprising ethanol, water and sodium ethoxide (10 ml) were heated at 333 K with continuous stirring for 6 h. The mixture was kept aside for slow evaporation. After a week, crystals suitable for X-ray diffraction were obtained.

### Refinement

H atoms were positioned geometrically [C—H = 0.95 (aromatic) or 0.99 Å (methylene); O—H= 0.82–0.85 Å] and refined using a riding model, with *U*_iso_(H) = 1.2-1.5*U*_eq_(C,O).

### Crystal data

**Table:**

C_13_H_11_N_3_O·H_2_O	*F*(000) = 512
*M**_r_* = 243.26	*D*_x_ = 1.321 Mg m^−^^3^
Orthorhombic, *P**n**a*2_1_	Mo *K*α radiation, λ = 0.71073 Å
Hall symbol: P 2c -2n	Cell parameters from 1403 reflections
*a* = 12.556 (5) Å	θ = 2.5–20.2°
*b* = 20.881 (8) Å	µ = 0.09 mm^−^^1^
*c* = 4.6651 (18) Å	*T* = 173 K
*V* = 1223.1 (8) Å^3^	Needle, colourless
*Z* = 4	0.40 × 0.05 × 0.04 mm

### Data collection

**Table:**

Bruker SMART APEXII diffractometer	1118 reflections with *I* > 2σ(*I*)
Radiation source: sealed tube	*R*_int_ = 0.132
Graphite monochromator	θ_max_ = 28.2°, θ_min_ = 1.9°
CCD scan	*h* = −16→16
15911 measured reflections	*k* = −27→27
1677 independent reflections	*l* = −6→6

### Refinement

**Table:**

Refinement on *F*^2^	Secondary atom site location: difference Fourier map
Least-squares matrix: full	Hydrogen site location: inferred from neighbouring sites
*R*[*F*^2^ > 2σ(*F*^2^)] = 0.052	H-atom parameters constrained
*wR*(*F*^2^) = 0.140	*w* = 1/[σ^2^(*F*_o_^2^) + (0.0825*P*)^2^] where *P* = (*F*_o_^2^ + 2*F*_c_^2^)/3
*S* = 0.98	(Δ/σ)_max_ < 0.001
1677 reflections	Δρ_max_ = 0.19 e Å^−^^3^
164 parameters	Δρ_min_ = −0.24 e Å^−^^3^
1 restraint	Extinction correction: *SHELXL97* (Sheldrick, 2008), Fc^*^=kFc[1+0.001xFc^2^λ^3^/sin(2θ)]^-1/4^
Primary atom site location: structure-invariant direct methods	Extinction coefficient: 0.038 (6)

### Special details

**Table:**

Geometry. All esds (except the esd in the dihedral angle between two l.s. planes) are estimated using the full covariance matrix. The cell esds are taken into account individually in the estimation of esds in distances, angles and torsion angles; correlations between esds in cell parameters are only used when they are defined by crystal symmetry. An approximate (isotropic) treatment of cell esds is used for estimating esds involving l.s. planes.
Refinement. Refinement of F^2^ against ALL reflections. The weighted R-factor wR and goodness of fit S are based on F^2^, conventional R-factors R are based on F, with F set to zero for negative F^2^. The threshold expression of F^2^ > 2sigma(F^2^) is used only for calculating R-factors(gt) etc. and is not relevant to the choice of reflections for refinement. R-factors based on F^2^ are statistically about twice as large as those based on F, and R- factors based on ALL data will be even larger.

### Fractional atomic coordinates and isotropic or equivalent isotropic displacement parameters (Å^2^)

**Table:**

	*x*	*y*	*z*	*U*_iso_*/*U*_eq_	
N1	0.3160 (2)	0.27237 (12)	0.6604 (7)	0.0335 (7)	
N2	0.4106 (2)	0.25008 (13)	0.5616 (7)	0.0372 (7)	
N3	0.4253 (2)	0.19473 (13)	0.6976 (8)	0.0389 (8)	
C4	0.3429 (3)	0.18041 (15)	0.8807 (9)	0.0343 (8)	
C5	0.3241 (3)	0.12821 (16)	1.0631 (10)	0.0455 (10)	
H5	0.3720	0.0931	1.0769	0.055*	
C6	0.2315 (3)	0.13125 (18)	1.2207 (10)	0.0524 (11)	
H6	0.2153	0.0974	1.3495	0.063*	
C7	0.1594 (3)	0.18339 (18)	1.1969 (10)	0.0464 (9)	
H7	0.0962	0.1829	1.3089	0.056*	
C8	0.1776 (3)	0.23429 (16)	1.0183 (8)	0.0380 (9)	
H8	0.1293	0.2692	1.0038	0.046*	
C9	0.2720 (3)	0.23174 (14)	0.8583 (8)	0.0301 (8)	
C10	0.2800 (3)	0.33671 (14)	0.5809 (9)	0.0344 (8)	
H10A	0.2019	0.3360	0.5514	0.041*	
H10B	0.3137	0.3492	0.3974	0.041*	
C11	0.3069 (3)	0.38601 (15)	0.8068 (8)	0.0310 (8)	
C12	0.2259 (3)	0.42481 (15)	0.9188 (9)	0.0364 (8)	
H12	0.1545	0.4193	0.8558	0.044*	
C13	0.2499 (3)	0.47148 (15)	1.1226 (9)	0.0419 (9)	
H13	0.1948	0.4979	1.1972	0.050*	
C14	0.3532 (3)	0.47944 (16)	1.2161 (10)	0.0423 (9)	
H14	0.3693	0.5115	1.3539	0.051*	
C15	0.4335 (3)	0.44073 (16)	1.1095 (9)	0.0396 (9)	
H15	0.5044	0.4458	1.1767	0.048*	
C16	0.4107 (3)	0.39448 (16)	0.9047 (9)	0.0377 (8)	
H16	0.4663	0.3684	0.8308	0.045*	
O17	0.51045 (19)	0.16046 (12)	0.6496 (9)	0.0576 (10)	
O1W	0.4934 (2)	0.03436 (11)	0.4506 (7)	0.0472 (7)	
H1W	0.4943	0.0098	0.5883	0.071*	
H2W	0.4986	0.0728	0.5103	0.071*	

### Atomic displacement parameters (Å^2^)

**Table:**

	*U*^11^	*U*^22^	*U*^33^	*U*^12^	*U*^13^	*U*^23^
N1	0.0399 (15)	0.0262 (13)	0.0344 (17)	0.0007 (12)	−0.0016 (14)	0.0040 (13)
N2	0.0383 (15)	0.0306 (13)	0.0426 (18)	0.0002 (13)	0.0004 (15)	−0.0061 (14)
N3	0.0389 (15)	0.0276 (13)	0.0503 (19)	0.0033 (12)	−0.0076 (16)	−0.0087 (15)
C4	0.0400 (18)	0.0240 (15)	0.039 (2)	−0.0013 (14)	−0.0091 (18)	−0.0034 (16)
C5	0.063 (2)	0.0263 (16)	0.048 (2)	−0.0027 (17)	−0.021 (2)	0.0027 (18)
C6	0.080 (3)	0.034 (2)	0.043 (2)	−0.017 (2)	−0.018 (3)	0.0126 (19)
C7	0.056 (2)	0.043 (2)	0.039 (2)	−0.0120 (17)	−0.003 (2)	0.008 (2)
C8	0.044 (2)	0.0316 (18)	0.038 (2)	−0.0022 (15)	−0.0036 (18)	0.0001 (16)
C9	0.0396 (18)	0.0241 (14)	0.0268 (19)	−0.0015 (13)	−0.0043 (16)	0.0025 (14)
C10	0.045 (2)	0.0259 (15)	0.0321 (19)	0.0012 (14)	−0.0032 (17)	0.0053 (15)
C11	0.0410 (18)	0.0214 (14)	0.0304 (18)	−0.0046 (14)	−0.0022 (16)	0.0067 (14)
C12	0.0353 (17)	0.0297 (15)	0.044 (2)	0.0020 (14)	−0.0066 (18)	0.0010 (17)
C13	0.0461 (19)	0.0287 (16)	0.051 (3)	0.0061 (15)	−0.005 (2)	−0.0025 (18)
C14	0.056 (2)	0.0299 (16)	0.041 (2)	−0.0108 (16)	−0.001 (2)	−0.0005 (18)
C15	0.0401 (19)	0.0376 (17)	0.041 (2)	−0.0109 (16)	−0.0017 (18)	0.0010 (17)
C16	0.0376 (18)	0.0372 (17)	0.038 (2)	0.0002 (15)	0.0028 (17)	0.0048 (17)
O17	0.0404 (14)	0.0367 (13)	0.096 (3)	0.0084 (11)	−0.0063 (17)	−0.0224 (18)
O1W	0.0745 (18)	0.0287 (12)	0.0382 (15)	−0.0030 (12)	−0.0035 (14)	−0.0018 (12)

### Geometric parameters (Å, º)

**Table:**

N1—N2	1.356 (4)	C10—H10A	0.9900
N1—C9	1.370 (4)	C10—H10B	0.9900
N1—C10	1.465 (4)	C11—C16	1.393 (5)
N2—N3	1.331 (4)	C11—C12	1.401 (5)
N3—O17	1.306 (4)	C12—C13	1.394 (5)
N3—C4	1.374 (5)	C12—H12	0.9500
C4—C9	1.398 (4)	C13—C14	1.379 (5)
C4—C5	1.403 (5)	C13—H13	0.9500
C5—C6	1.377 (6)	C14—C15	1.385 (5)
C5—H5	0.9500	C14—H14	0.9500
C6—C7	1.421 (6)	C15—C16	1.389 (5)
C6—H6	0.9500	C15—H15	0.9500
C7—C8	1.370 (5)	C16—H16	0.9500
C7—H7	0.9500	O1W—H1W	0.8225
C8—C9	1.401 (5)	O1W—H2W	0.8519
C8—H8	0.9500		
			
N2—N1—C9	111.7 (3)	N1—C10—C11	112.3 (3)
N2—N1—C10	119.9 (3)	N1—C10—H10A	109.1
C9—N1—C10	127.8 (3)	C11—C10—H10A	109.1
N3—N2—N1	104.9 (3)	N1—C10—H10B	109.1
O17—N3—N2	120.5 (3)	C11—C10—H10B	109.1
O17—N3—C4	127.1 (3)	H10A—C10—H10B	107.9
N2—N3—C4	112.4 (3)	C16—C11—C12	118.9 (3)
N3—C4—C9	105.4 (3)	C16—C11—C10	121.6 (3)
N3—C4—C5	132.3 (3)	C12—C11—C10	119.5 (3)
C9—C4—C5	122.3 (3)	C13—C12—C11	120.1 (3)
C6—C5—C4	115.5 (3)	C13—C12—H12	119.9
C6—C5—H5	122.3	C11—C12—H12	119.9
C4—C5—H5	122.3	C14—C13—C12	120.2 (3)
C5—C6—C7	122.1 (4)	C14—C13—H13	119.9
C5—C6—H6	118.9	C12—C13—H13	119.9
C7—C6—H6	118.9	C13—C14—C15	120.1 (4)
C8—C7—C6	122.4 (4)	C13—C14—H14	120.0
C8—C7—H7	118.8	C15—C14—H14	120.0
C6—C7—H7	118.8	C14—C15—C16	120.2 (3)
C7—C8—C9	115.9 (3)	C14—C15—H15	119.9
C7—C8—H8	122.1	C16—C15—H15	119.9
C9—C8—H8	122.1	C15—C16—C11	120.5 (3)
N1—C9—C4	105.5 (3)	C15—C16—H16	119.8
N1—C9—C8	132.6 (3)	C11—C16—H16	119.8
C4—C9—C8	121.9 (3)	H1W—O1W—H2W	109.4
			
C9—N1—N2—N3	0.5 (4)	C5—C4—C9—N1	−179.4 (3)
C10—N1—N2—N3	172.9 (3)	N3—C4—C9—C8	−179.3 (3)
N1—N2—N3—O17	179.9 (3)	C5—C4—C9—C8	0.3 (5)
N1—N2—N3—C4	0.2 (4)	C7—C8—C9—N1	179.4 (4)
O17—N3—C4—C9	179.6 (3)	C7—C8—C9—C4	−0.1 (5)
N2—N3—C4—C9	−0.8 (4)	N2—N1—C10—C11	−96.1 (4)
O17—N3—C4—C5	0.0 (7)	C9—N1—C10—C11	74.9 (4)
N2—N3—C4—C5	179.6 (4)	N1—C10—C11—C16	54.8 (4)
N3—C4—C5—C6	178.9 (4)	N1—C10—C11—C12	−126.0 (3)
C9—C4—C5—C6	−0.6 (5)	C16—C11—C12—C13	0.7 (5)
C4—C5—C6—C7	0.8 (6)	C10—C11—C12—C13	−178.6 (3)
C5—C6—C7—C8	−0.7 (6)	C11—C12—C13—C14	−0.4 (6)
C6—C7—C8—C9	0.3 (6)	C12—C13—C14—C15	−0.5 (6)
N2—N1—C9—C4	−1.0 (4)	C13—C14—C15—C16	1.0 (6)
C10—N1—C9—C4	−172.6 (3)	C14—C15—C16—C11	−0.7 (6)
N2—N1—C9—C8	179.5 (4)	C12—C11—C16—C15	−0.1 (5)
C10—N1—C9—C8	7.8 (6)	C10—C11—C16—C15	179.1 (3)
N3—C4—C9—N1	1.0 (4)		

### Hydrogen-bond geometry (Å, º)

Cg1 is the centroid of the C11–C16 phenyl ring.

**Table:**

*D*—H···*A*	*D*—H	H···*A*	*D*···*A*	*D*—H···*A*
O1*W*—H1*W*···O1*W*^i^	0.82	1.93	2.744 (3)	169
O1*W*—H2*W*···O17	0.85	1.95	2.800 (3)	180
C10—H10*A*···O17^ii^	0.99	2.45	3.400 (5)	161
C10—H10*B*···*Cg*3^iii^	0.99	2.51	3.382 (4)	147

Symmetry codes: (i) −*x*+1, −*y*, *z*+1/2; (ii) *x*−1/2, −*y*+1/2, *z*; (iii) *x*, *y*, *z*−1.
